# Potential pathways to the onset and development of eating disorders in people with overweight and obesity: A scoping review

**DOI:** 10.1111/obr.13840

**Published:** 2024-10-04

**Authors:** Rabia Khalid, Natalie B. Lister, Susan J. Paxton, Sarah Maguire, Sol Libesman, Anna L. Seidler, Kelly Cooper, Fiona Quigley, Jacqlyn Yourell, Louise A. Baur, Hiba Jebeile

**Affiliations:** ^1^ Children's Hospital Westmead Clinical School The University of Sydney Westmead New South Wales Australia; ^2^ Charles Perkins Centre The University of Sydney Sydney New South Wales Australia; ^3^ School of Psychology and Public Health La Trobe University Melbourne Victoria Australia; ^4^ InsideOut Institute for Eating Disorders, Charles Perkins Centre The University of Sydney Sydney New South Wales Australia; ^5^ National Health and Medical Research Council Clinical Trials Centre The University of Sydney Sydney New South Wales Australia; ^6^ Weight Issues Network Sydney New South Wales Australia; ^7^ Institute of Nursing and Health Research Ulster University Belfast UK; ^8^ Department of Family, Youth and Community Sciences University of Florida College of Agricultural and Life Sciences Gainesville, Florida USA; ^9^ Fit Minded, Inc Phoenix Arizona USA; ^10^ Weight Management Services The Children's Hospital at Westmead Westmead New South Wales Australia

**Keywords:** disordered eating, eating disorder pathways, obesity treatment, predictors, risk factors, weight management

## Abstract

**Objective:**

To describe pathways to eating disorder (ED) development that have been evaluated in people with overweight and obesity.

**Methods:**

Four databases were searched to identify studies testing ED development models in adolescents (10–19 years) or adults (>19 years) with overweight and obesity. Explanatory variables were thematically grouped into constructs to describe pathways to each ED outcome.

**Results:**

Of 2226 studies screened, 46 (10 adolescent; 36 adult) were included. Study samples were predominantly female, ranging from 22 to 2236 participants and mean age 12.3 to 56.0 years. In total, 207 explanatory variables were grouped into 18 constructs to summarize 107 pathways that were identified. The most common ED outcome was binge eating (*n* = 24 studies), followed by global ED psychopathology (*n* = 10 studies). Across pathways to ED development, negative affect was the most proposed construct, followed by preoccupation with weight/shape and weight stigma.

**Conclusion:**

Pathways to ED development in people with overweight and obesity are complex and may include more than 18 different explanatory factors of which negative affect, preoccupation with weight/shape, and weight stigma are the most common. More research on adolescents, males, and the spectrum of ED in diverse populations is required for early identification and intervention.

## 1 INTRODUCTION

People with higher weight are more likely to have eating disorders (EDs) or disordered eating behaviors compared to those classified as having underweight or a normal weight.^1^, ^2^ Co‐existing overweight and ED behaviors are suggested to be increasing at a faster rate than either condition alone.^3^, ^4^ However, the rate of diagnosis, referral for care, and treatment of EDs is lower in people with overweight and obesity, despite losing a larger proportion of body weight or having similar illness duration to those with under, or normal weight classification.^5^, ^6^ Aside from being at greater risk of having their ED untreated, people with overweight and obesity are also more likely to seek or be prescribed weight management therapy.^7^, ^8^ To improve early identification, prevention, and the safety and efficacy of treatment for both conditions, it is important to understand the risk and protective pathways to ED development specific to people with overweight or obesity.^9^

The development of EDs involves a complex combination of biological, genetic, environmental, and sociocultural factors. Biological factors encompass neurodevelopmental and neurobiological attributes which form the basis of personality and cognitive traits.^10^ Certain forms of genetic‐based temperament are ED risk factors such as perfectionism, harm avoidance, dietary preoccupation, and altered interoceptive awareness.^10^ Sociocultural factors refer to a culture's ideology communicated through socialization agents, for example, cultural ideals of body and attractiveness perpetuated through parents, peers, and media.^10^, ^11^ On the other hand, environmental determinants of ED risk refers to external influences (e.g., trauma, nutrition, etc.) on a person's psychology and gene expression.^10^ It is argued that given the widespread prevalence of sociocultural and environmental triggers for ED risk, in contrast to the much lower occurrence of the disease, biology and genetic temperament play a core role in its development.^10^ However, there is vast empirical evidence for various sociocultural and environmental risk factors of EDs which are more likely to be modifiable and therefore play an important role in prevention.^10^, ^12^, ^13^

It is possible that the biological and genetic risk profile and responses to sociocultural and environmental contributors to EDs in people with overweight and obesity may differ to people with a lower weight.^9^, ^14^ Genetic temperament and psychological traits associated with body dissatisfaction, along with sociocultural and environmental factors such as pressure regarding appearance and weight‐related teasing and bullying, are suggested to be more prevalent in those with overweight and obesity compared to those with a lower weight.^15^, ^16^, ^17^, ^18^ In individuals with diagnosed binge ED, other disordered eating behaviors such as skipping meals, excessive exercise, and restricting specific foods for weight control purposes have been observed to be lower in people with overweight and obesity compared to those with lower weight.^19^ Similarly, in a sample of women with clinical or subclinical binge ED with similar levels of ED symptomatology, those with obesity had lower scores of dietary restraint.^20^ Given the potential difference in risk factors, which form the foundation of understanding mechanisms and pathways to disease development, models of ED development should be specific to people with overweight and obesity.

Models of disease aetiology consist of pathways outlining inter‐relationships between variables such as risk and protective factors. There has been extensive research on models of potential pathways to the development of various EDs, although prevention and treatment remain difficult.^21^ In a 2016 review, 54 ED models were identified, and 23 have led to intervention development.^21^ Common risk factors across models include preoccupation with weight and shape, negative affect, self‐esteem deficits, and interpersonal issues.^21^ However, people with overweight or obesity were poorly represented in these ED models. Models describing ED development, particularly those for anorexia nervosa, are often tested among people with a lower weight. Of the well‐known ED development models, to our knowledge only the dual pathway model^22^ has been tested and compared for its applicability to people with lower versus those with overweight and obesity.^23^ The original dual pathway model suggests that body dissatisfaction because of internalization of thin body ideals leads to negative affect and dietary restraint which results in bulimic and binge eating.^22^ The study by Welsh et al. (2016) found that this model was only partially applicable to those with overweight and obesity with binge ED.^23^ Dietary restraint was not found to mediate the model's pathway between body dissatisfaction and bulimic symptoms in the sample with overweight and obesity; however, it was supported in those with a “healthy” weight. Similarly, in a young community sample of men, the pathway from body image concerns to loss‐of‐control eating through dietary restraint was observed to be greater in men classified as having low‐to‐normal body mass index (BMI) compared to those classified as having overweight or obesity.^24^ These variations based on weight status further reinforce the need to ensure that ED development models are applicable to people with overweight and obesity. Therefore, the aim of this scoping review was (a) to identify and describe pathways to ED development that have been tested or evaluated in adolescents or adults with overweight and obesity, and (b) summarize the most commonly proposed variables forming pathways in identified models.

## 2 METHODS

This scoping review was conducted in accordance with the JBI methodology for scoping reviews^25^ and the Preferred Reporting Items for Systematic Reviews and Meta‐Analyses extension for Scoping Reviews (PRISMA‐ScR) guidance.^26^ A protocol for this review was prospectively uploaded on Open Science Framework.^27^

### 2.1 Eligibility criteria

#### 2.1.1 Participants

The participants in eligible studies for this review were adolescents (aged 10–19 years) or adults (≥19 years) with overweight or obesity. Overweight and obesity were defined as a BMI greater than 25 and 30 kg/m^2^, respectively in adults. For adolescents, overweight and obesity were defined as greater than the 85th and 95th percentiles on US Centers for Disease Control growth charts, respectively, or as the adult equivalent BMI. Publications reporting on participants with varied weight statuses (i.e., not exclusive to overweight or obesity) were excluded unless data were reported separately for those with overweight and obesity.

#### 2.1.2 Concept

In this review, we included sources of evidence that evaluated explicit conceptual models or frameworks describing the pathways and relationship between at least two independent variables (other than weight status) and the development of ED behaviors or an ED (i.e., the outcome) in adolescents and/or adults with overweight or obesity. Individual predictors have previously been explored;^12^, ^13^, ^28^ therefore, the inclusion of paths between a minimum of two independent variables was required to meet this review's aim of exploring possible pathways to ED development. Possible outcomes included binge ED or behaviors, bulimia nervosa, anorexia nervosa, fasting, dietary restraint, laxative abuse (for the purpose of weight loss), purging, excessive exercise, and/or other eating psychopathology.

#### 2.1.3 Context

Contextual focus was directed to studies that tested or evaluated the applicability of the model pathways to the development of EDs or ED behaviors and symptoms in adolescents and/or adults with overweight or obesity. Testing could involve quantitative models and statistical analyses or qualitative interviews.

#### 2.1.4 Types of sources

Sources of evidence considered for this review included peer‐reviewed journal articles, with no restrictions on study methodology, including quantitative, qualitative, or mixed‐methods study designs. Systematic and scoping reviews were excluded after being reviewed for relevant studies. Studies published in a language other than English were also excluded. No date restrictions were placed on eligible sources of information.

### 2.2 Search strategy

The databases MEDLINE, PsycINFO, Embase, and Google Scholar were searched to 25 July 2022 to identify potential studies for inclusion. The search strategy, including keywords and index terms, was determined through discussion with the research team and by scanning background literature. The search strategy, adapted for each database, can be found in Table S1. The reference list of all eligible sources of information was checked for further eligible studies.

### 2.3 Study/source of evidence selection

All sources of information or studies were uploaded into Covidence for screening and source selection.^29^ Title and abstracts of studies were screened for inclusion independently by two reviewers. Full texts of studies were screened by one reviewer and verified by a second reviewer. Disagreements in screening were reviewed and resolved by discussion and, if required, by the decision of a third reviewer.

### 2.4 Data extraction

One author independently extracted data using a study specific data extraction form, and a second reviewed the extraction.^27^ Extracted data consisted of information on study population demographics, type(s) of ED and measurement tool used and descriptions of explanatory variables, and directions of pathways in identified conceptual models.

### 2.5 Data analysis and presentation

A narrative synthesis of included models was conducted in accordance with the Synthesis Without Meta‐analysis (SWiM) guidelines (when applicable).^30^ Based on key themes identified across the explanatory variables of model pathways, constructs were developed and allocated. These constructs were used to describe the pathways to the development of ED outcomes. Pathway results were synthesized separately for each ED outcome identified and for key subgroups (adults versus adolescents and those seeking versus not seeking obesity treatment). Consultation with individuals with lived experience of both overweight and obesity and EDs was also undertaken for feedback on our findings. Where reported, variance accounted for by the models (*R* ^2^) has been summarized to assist with interpretation of results. However, models included in this review vary widely in their complexity, so *R* ^2^ should not be used to compare goodness of fit between models. It is likely that more complex models include parameters to account for a great degree of variance. Additionally, most data are cross‐sectional and susceptible to bias,^31^, ^32^ therefore, these data should be interpreted with caution. We descriptively summarized the quantitative findings of any causal models based on longitudinal data.

## 3 RESULTS

### 3.1 Included studies

A total of 2226 studies were identified in the search, of which 46 met the inclusion criteria (Figure S1). Characteristics of included studies are described in Table S2. The majority of the included studies were conducted in adult populations (36 studies)^23^, ^24^, ^33^, ^34^, ^35^, ^36^, ^37^, ^38^, ^39^, ^40^, ^41^, ^42^, ^43^, ^44^, ^45^, ^46^, ^47^, ^48^, ^49^, ^50^, ^51^, ^52^, ^53^, ^54^, ^55^, ^56^, ^57^, ^58^, ^59^, ^60^, ^61^, ^62^, ^63^, ^64^, ^65^, ^66^ of which 25 focused on obesity treatment‐seeking populations.^34^, ^37^, ^38^, ^39^, ^40^, ^41^, ^43^, ^44^, ^45^, ^48^, ^49^, ^50^, ^51^, ^53^, ^54^, ^55^, ^56^, ^57^, ^58^, ^59^, ^60^, ^62^, ^63^, ^64^, ^65^ Of the 10 adolescent studies,^67^, ^68^, ^69^, ^70^, ^71^, ^72^, ^73^, ^74^, ^75^, ^76^ five focused on obesity treatment seeking populations,^67^, ^68^, ^69^, ^70^, ^76^ whereas two included a mixture of both those seeking and not seeking obesity treatment.^71^, ^72^ Study sample sizes and mean age ranged from 22 to 2236 and 12.3 to 56 years, respectively. Samples were predominantly females (sex) and White or Caucasian (ethnicity). The average adult equivalent BMI ranged from 27.09 to 32.89 kg/m^2^ for adolescents and 25.4 to 50.8 kg/m^2^ for adults. All identified studies used quantitative methods to test ED development models in people with overweight and obesity, and only three studies were longitudinal^36^, ^64^, ^75^ with the remaining being cross‐sectional.

### 3.2 ED outcomes

The most common ED outcome measured was binge eating (24 studies),^33^, ^35^, ^37^, ^38^, ^39^, ^40^, ^42^, ^45^, ^46^, ^48^, ^49^, ^50^, ^52^, ^53^, ^56^, ^58^, ^60^, ^61^, ^62^, ^63^, ^64^, ^65^, ^66^, ^67^ followed by global ED psychopathology (10 studies),^43^, ^44^, ^46^, ^47^, ^51^, ^55^, ^68^, ^69^, ^73^, ^76^ loss of control eating (eight studies),^24^, ^41^, ^42^, ^51^, ^54^, ^59^, ^71^, ^72^ dietary restraint (six studies),^23^, ^34^, ^57^, ^70^, ^74^, ^75^ and bulimia symptoms or behaviors (three studies).^23^, ^53^, ^75^ Most studies used the Binge Eating Scale (BES) to measure binge eating, while the Eating Disorder Examination Questionnaire (EDE‐Q) was commonly used to obtain global ED psychopathology.

### 3.3 Model constructs

**FIGURE 1:**
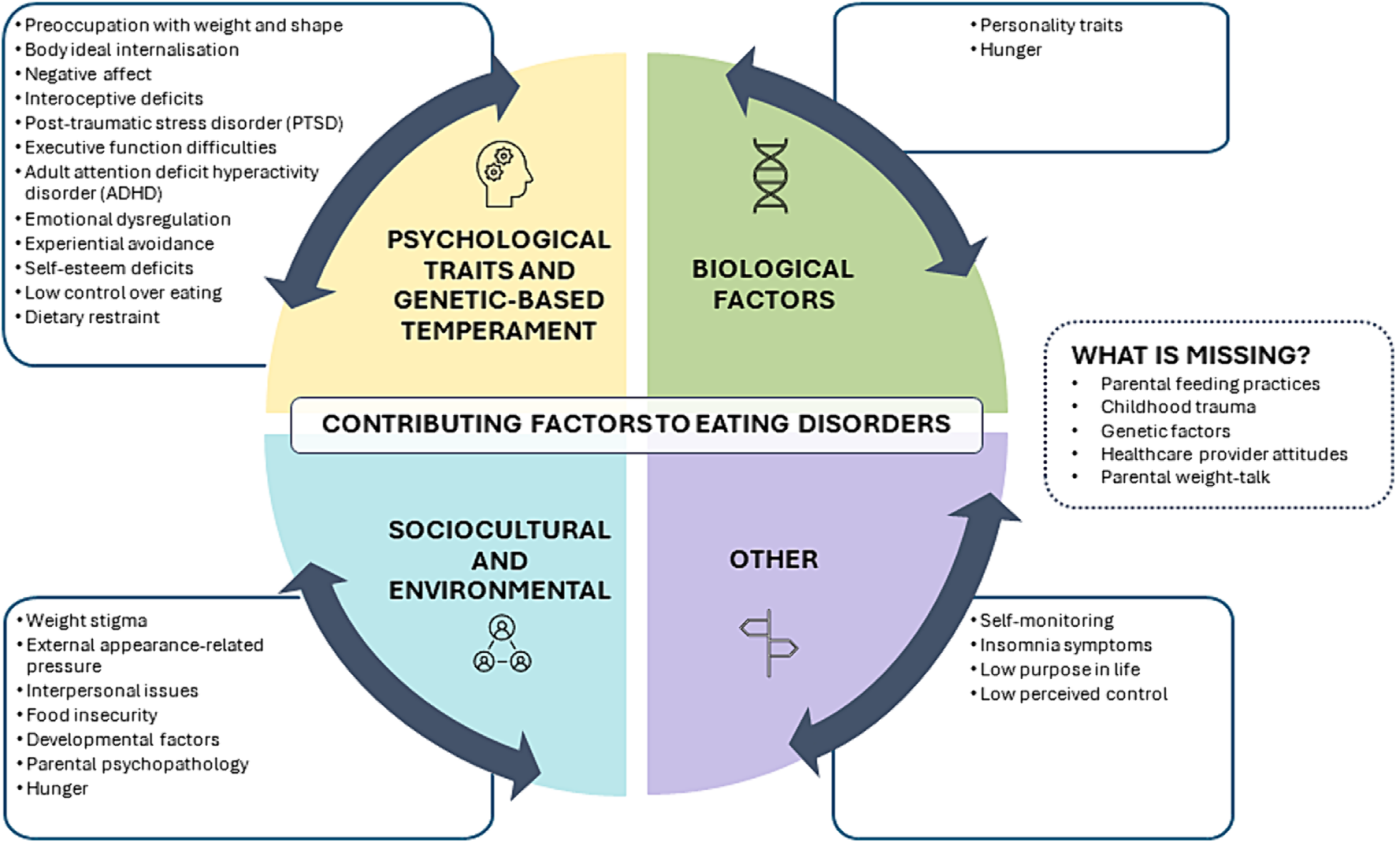
Interplay of factors contributing to the aetiology of eating disorders. *Note*: Constructs listed in each category are those which have been identified in this review, and those listed as missing have been proposed through stakeholder consultation.

Across models, a total of 207 explanatory variables were observed to form 107 pathways to the development of ED outcomes. These variables were categorized into 18 constructs based on common themes, with an “Other” category for uncommon variables that did not match the themes of other constructs, which are defined in Table 1. Negative affect was the most commonly observed construct, with 29 variables (16.3%) across 41.7% of adult models and seven variables (24.1%) in 40% of adolescent models. This was followed by weight stigma for adults (18 variables, 10.1%) and preoccupation with weight and shape for adolescents (six variables, 20.7%). Other constructs reported in both adult and adolescent studies included dietary restraint, emotion regulation, interoceptive deficits, external appearance‐related pressure, and self‐esteem deficits. Most of the identified constructs can be considered psychological traits and genetic‐based temperament or sociocultural and environmental factors contributing to EDs (Figure 1).

**TABLE 1:** Summary of explanatory variables and corresponding constructs observed in models.

			Adult models		Adolescent models	
Construct	Construct definition	Independent variables identified in studies	Variable frequency *n* (%)Total = 178	Studies *n* (%)Total = 36	Variable frequency *n* (%)Total = 29	Studies *n* (%)Total = 10
Dietary restraint	Cognitive effort to restrict food intake, or the intent to restrict food intake	Dietary restraint Restraint Food thought suppression Restraint intention Food‐related willingness (measure of ability to control restrain from eating despite cravings) Cognitive restraint	12 (6.7%	6 (16.7)	2 (6.9)	2 (20.0)
Emotion dysregulation	Difficulties in regulating emotion	Difficulties with impulse control	14 (7.9%)	6 (16.7)	1 (3.4)	2 (20.0)
		Difficulties in emotion regulation				
		Negative urgency				
		Impulse dysregulation				
		Emotion dysregulation				
		Emotional eating				
Interoceptive deficits	Difficulties in perceiving and interpreting internal bodily sensations (e.g., emotions)	Interoceptive deficits Difficulty in describing feelings Difficulty in identifying feelings Externally oriented thinking Alexithymia Perception of threat from emotions	7 (3.9%)	4 (11.1)	2 (6.9)	1 (10.0)
Personality traits	A person's characteristic patterns of thoughts, feelings, and behaviors	Low extraversion Low openness Reduced emotional expressiveness Low conscientiousness High neuroticism Low agreeableness	14 (7.9)	2 (5.6)	0 (0.0)	0 (0.0)
Experiential avoidance	An attempt or desire to suppress unwanted internal experiences	Distraction orientated coping Weight‐related experiential avoidance or psychological inflexibility General experiential avoidance Negative reinforcement eating expectancies (belief that eating will help to mitigate distress)	9 (5.1)	6 (16.7)	0 (0.0)	0 (0.0)
External pressure	Appearance‐related pressure from external sources	Family pressure Media pressure Peer pressure Pressure to be thin Perceived friend influence Perceptions of teasing	3 (1.7)	2 (5.6)	2 (6.9)	2 (20.0)
Interpersonal issues	Difficulties or conflicts in relationships or interactions with other others	Interpersonal problems Couple dissatisfaction (Marriage/relationship quality) Interpersonal problems Avoidant attachment Anxious attachment	6 (3.4)	4 (11.1)	0 (0.0)	0 (0.0)
Negative affect	Negative emotional states or feelings of emotional distress	Anxiety or anxiety symptoms Negative (eating‐related) moral emotion state (disgust, guilt, shame) Negative affect or overall depressive mood Sadness Negative affective experiences Weight‐related negative affect Depression or depressive symptoms Stress Daily hassles (measure of stress from daily hassles)	29 (16.3)	15 (41.7)	7 (24.1)	4 (40.0)
Preoccupation with weight and shape	Persistent, repetitive, and intrusive thoughts about one's body weight and shape, with these thoughts playing an overly important role in one's perceived self‐worth.	Fear of negative appearance evaluation Body dissatisfaction Overvaluation of shape/weight Appearance evaluation Overweight preoccupation Weight‐related social comparison Feeling fat Body image Bodily shame Body esteem	24 (13.5)	8 (22.2)	6 (20.7)	5 (50.0)
Self‐esteem deficits	Negative appraisal of one's own self‐worth and value as a person	Low self‐esteem High self‐criticism High self‐disgust Low self‐compassion Low reassured self	6 (3.4)	4 (11.1)	4 (13.8)	3 (30.0)
Weight stigma	Negative beliefs, attitudes, stereotypes, and discriminatory behaviors towards people with overweight and obesity	Self‐devaluation (internalized weight stigma)	18 (10.1)	9 (0.25)	2 (6.9)	1 (10.0)
		Weight‐related reassured self				
		Weight‐related external shame				
		Weight‐related hated or inadequate self				
		Weight self‐stigma				
		Weight bias internalization				
		Stigma consciousness				
		Weight discrimination				
		Weight stigma				
Low control over eating	Decreased sense of control over eating behaviors	Frequency of loss‐of‐control eating	9 (5.1)	6 (16.7)	0 (0.0)	0 (0.0)
		Disinhibition				
		Self‐efficacy for controlled eating				
		overeating				
Body ideal internalization	The degree to which an individual subscribes to socially reinforced ideals of weight and shape	Ideal muscular/athletic internalization	8 (4.5)	3 (8.3)	0 (0.0)	0 (0.0)
		Ideal thin/low body fat internalization				
		Fat concerns				
		Drive for muscularity				
Hunger	Physiological hunger	Hunger	1 (0.6)	2 (5.6)	0 (0.0)	0 (0.0)
Food insecurity	The condition of not having access to sufficient nutritious food to meet one's basic needs.	Food insecurity	0 (0.0)	2 (5.6)	0 (0.0)	0 (0.0)
Developmental factors	Factors impacting development during childhood	Negative life events	0 (0.0)	0 (0.0)	2 (6.9)	2 (20.0)
		Maternal parenting				
Parental psychopathology	Mental health of child's parents	Maternal psychopathology	0 (0.0)	0 (0.0)	1 (3.4)	1 (10.0)
Other		Low self‐monitoring (observing and processing one's own thoughts and behaviors)	18 (10.1)	7 (19.4)	0 (0.0)	0 (0.0)
		Insomnia symptoms				
		Adult attention deficit hyperactivity disorder (ADHD)				
		Low purpose in life (sense of meaning a person attributes to their existence)				
		Low perceived control (sense of control over one's life circumstances and behaviors)				
		Post‐traumatic stress disorder (PTSD) symptoms				
		Executive function difficulties (problems with cognitive processes required to complete tasks and solve problems)				

*Note*: Variable frequency refers to number of variables observed across models for each construct. Studies refers to number of studies examining variables for each construct.

In adolescents, the variance attributable to each construct was reported in three studies, with 24% of variance explained by depression, 11–14% by anxiety, and 39% by body shame (within the construct of preoccupation with weight and shape). For adults, emotional dysregulation accounted for 33–36% of the variance in two studies and an additional 2% when added to a model including depression and anxiety in another study. Negative affect varied between models accounting for 12–71% of the variance in seven studies. Preoccupation with weight and shape accounted for 30–69% of the variance in two studies and 6–16% in another study, where there was a lower proportion of variance explained for body dissatisfaction when depression was added to the model. Experiential avoidance accounted for 29–36% of variance in two studies, self‐esteem deficits 27–54% in three studies, weight stigma 36–45% in two studies, and low control over eating 17–21% in two studies. Variance attributable to body ideal internalization was reported in one study with 44% explained by muscle ideal internalization, 9% thin ideal internalization, and 18% drive for muscularity in men within the overweight category. Hunger accounted for 13–24% of the variance in two studies, and food insecurity was assessed with participant demographics in one study accounting for 10% of the variance for night eating and 7% for binge eating. Post‐traumatic stress disorder (PTSD) was reported as 25% in one study.

### 3.4 Model pathways

#### 3.4.1 Global ED psychopathology

**FIGURE 2:**
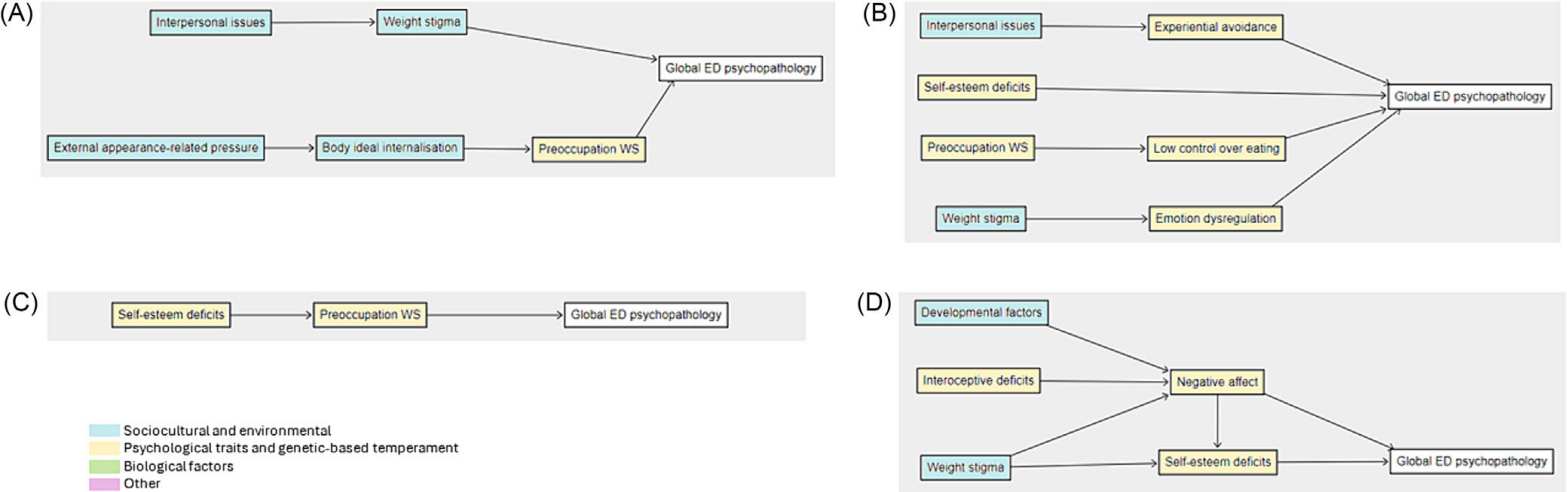
Pathways to global eating disorder psychopathology assessed in (A) adults not seeking obesity treatment (*n* = 2 studies), (B) adults seeking obesity treatment (*n* = 4 studies), (C) adolescents not seeking obesity treatment (*n* = 1 study), and (D) adolescents seeking obesity treatment (*n* = 3 studies). *Note*: Individual pathways from multiple studies have been collated to form figures; statistical significance was not considered, and complete represented model with all variables has not been tested together. Preoccupation WS = preoccupation with weight and shape; global ED psychopathology = global eating disorder psychopathology.

Two studies of non‐treatment seeking adults^46^, ^47^ proposed that the constructs interpersonal issues and external appearance‐related pressure may result in global ED psychopathology through weight stigma, body ideal internalization, and preoccupation with weight and shape (Figure 2A). Four studies focusing on treatment‐seeking adults^43^, ^44^, ^51^, ^55^ outlined that interpersonal issues, low self‐esteem deficits, preoccupation with weight and shape, and weight stigma may lead to global ED psychopathology through experiential avoidance, low control over eating, and emotion regulation (Figure 2B). One study assessed global ED psychopathology in non‐treatment seeking adolescents^73^; preoccupation with weight and shape was proposed to mediate the pathway between self‐esteem deficits and global ED psychopathology (Figure 2C). In three studies on treatment‐seeking adolescents,^68^, ^69^, ^76^ developmental factors, interoceptive deficits, and weight stigma were proposed to impact global ED psychopathology through negative affect and self‐esteem deficits (Figure 2D). In models with ED psychopathology as the outcome, the tested models accounted for 17% to 56% of symptoms in four studies. In one study, a model including self‐disgust and self‐compassion accounted for 51% of symptoms,^55^ and in another study, a model including external pressure, body ideal internalization, and preoccupation with weight and shape in overweight men accounted for 56% of symptoms.^47^

#### 3.4.2 Binge eating

**FIGURE 3:**
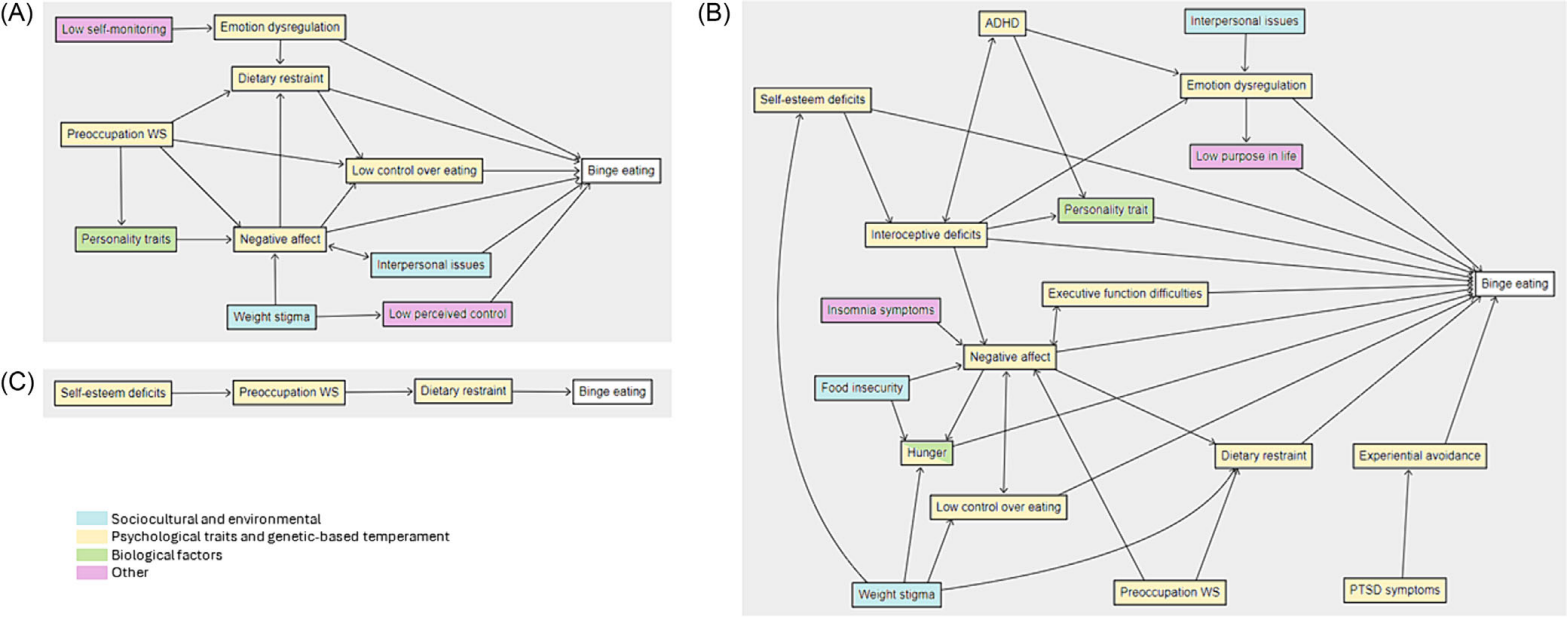
Pathways to binge eating assessed in (A) adults not seeking obesity treatment (*n* = 7 studies), (B) adults seeking obesity treatment (*n* = 16 studies), and (C) adolescents seeking obesity treatment (*n* = 1 study). *Note*: Individual pathways from multiple studies have been collated to form figures; statistical significance was not considered, and complete represented model with all variables has not been tested together. Preoccupation WS = preoccupation with weight and shape; ADHD = attention‐deficit/hyperactivity disorder; PTSD = post‐traumatic stress disorder.

In non‐treatment seeking adults, self‐monitoring, preoccupation with weight and shape, and weight stigma were theorized to lead to binge eating in seven studies (Figure 3A).^33^, ^35^, ^36^, ^42^, ^52^, ^61^, ^66^ These pathways were mediated by emotion regulation, dietary restraint, low control over eating, personality traits, negative affect, interpersonal issues, and low perceived control. In one study, some pathways between weight stigma and binge eating were significant for males but not females.^66^ Binge eating was assessed in 16 studies on obesity treatment‐seeking adults (Figure 3B).^37^, ^38^, ^39^, ^40^, ^45^, ^48^, ^49^, ^50^, ^53^, ^56^, ^58^, ^60^, ^62^, ^63^, ^64^, ^65^ Attention‐deficit/hyperactivity disorder (ADHD), insomnia symptoms, food insecurity, executive function difficulties, and PTSD symptoms were additional constructs proposed to lead to binge eating in this population. Mediators of these pathways included those hypothesized for non‐treatment seeking adults, along with hunger, self‐esteem deficits, experiential avoidance, interoceptive deficits, and low purpose in life. Among adolescents, one study assessed binge eating in an obesity treatment seeking population (Figure 3C),^67^ in which the pathway from self‐esteem deficits to binge eating through overvaluation of weight and shape and dietary restraint was evaluated.

In models with binge eating as the outcome, the tested models accounted for 11–47% of binge eating in 10 studies. In one study, the model examining the mediating effects of executive function and depression explained 46% of the variance in binge eating severity.^58^ In another study, the model including self‐esteem, interoceptive deficits, and impulse regulation explained 47% of the variance in binge eating.^37^

#### 3.4.3 Loss of control eating

**FIGURE 4:**
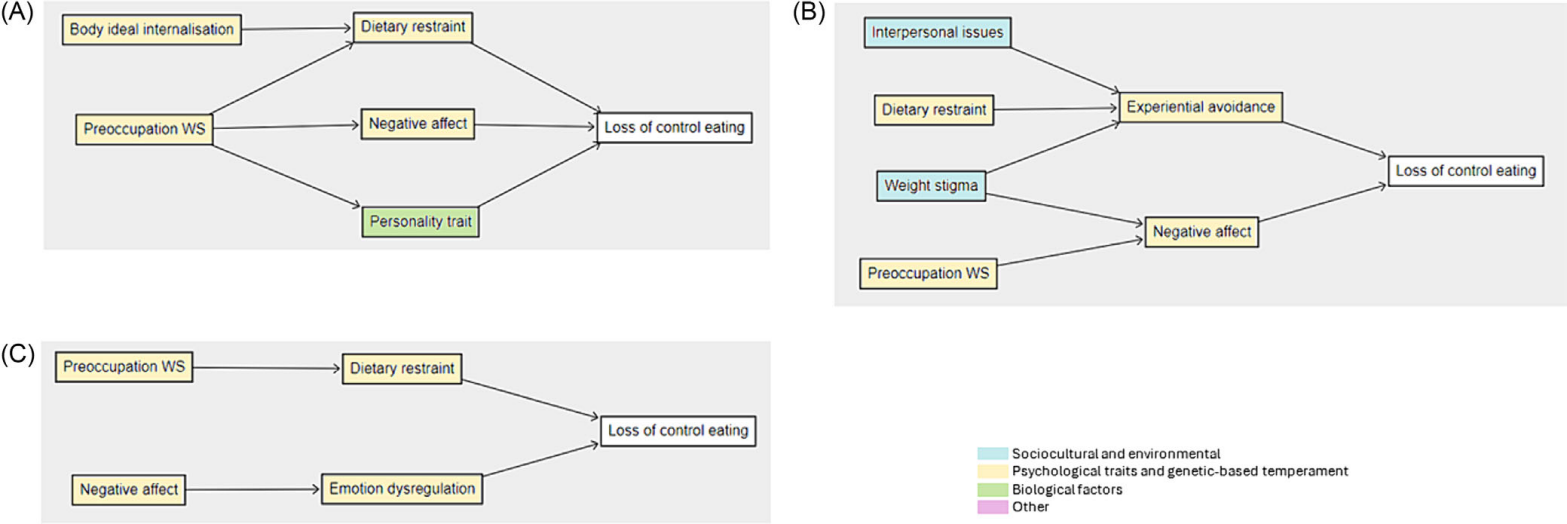
Pathways to loss of control eating assessed in (A) adults not seeking obesity treatment (*n* = 2 studies), (B) adults seeking obesity treatment (*n* = 4 studies), and (C) adolescents seeking obesity treatment (*n* = 2 studies). *Note*: Individual pathways from multiple studies have been collated to form figures; statistical significance was not considered, and complete represented model with all variables has not been tested together. Preoccupation WS = preoccupation with weight and shape.

Two studies assessed loss‐of‐control (LOC) eating in non‐treatment‐seeking adults.^24^, ^42^ Body ideal internalization and preoccupation with weight and shape were theorized to lead to LOC eating through dietary restraint, along with negative affect and personality traits (Figure 4A). Among studies of treatment‐seeking adults (*n* = 4 studies),^41^, ^51^, ^54^, ^59^ negative affect was also proposed to mediate the pathway between weight stigma and LOC eating (Figure 4B). Interpersonal issues, dietary restraint, and weight stigma were proposed to lead to LOC eating via experiential avoidance. Two studies assessed LOC eating in a mixed cohort of obesity treatment and non‐treatment‐seeking adolescents.^71^, ^72^ These studies reported potential pathways to LOC eating from preoccupation with weight and shape through dietary restraint, and from negative affect through emotion regulation (Figure 4C). In one study, a model including internalized weight stigma and experiential avoidance accounted for 30% of uncontrolled eating patterns.^55^

#### 3.4.4 Dietary restraint

**FIGURE 5:**
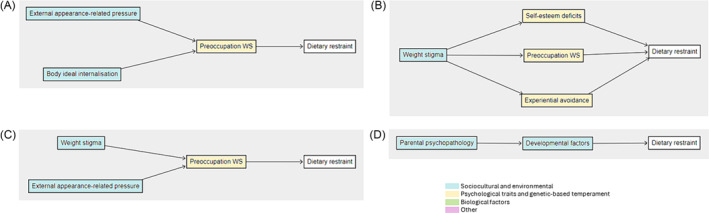
Pathways to dietary restraint assessed in (A) adults not seeking obesity treatment (*n* = 2 studies), (B) adults seeking obesity treatment (*n* = 2 studies), (C) adolescents not seeking obesity treatment (*n* = 2 studies), and (D) adolescents seeking obesity treatment (*n* = 1 study). *Note*: Individual pathways from multiple studies have been collated to form figures; statistical significance was not considered, and complete represented model with all variables has not been tested together. Preoccupation WS = preoccupation with weight and shape.

One study assessed dietary restraint as an outcome in non‐treatment‐seeking adults.^23^ External appearance‐related pressure and body ideal internalization were proposed to result in dietary restraint through preoccupation with weight and shape (Figure 5A). In studies on treatment seeking adults (*n* = 2),^34^, ^57^ weight stigma was proposed to lead to dietary restraint through self‐esteem deficits, preoccupation with weight and shape, and experiential avoidance (Figure 5B). Two studies on non‐treatment‐seeking adolescents^74^, ^75^ outlined weight stigma and external appearance‐related pressure as resulting in dietary restrain through preoccupation with weight and shape (Figure 5C). One study in obesity treatment‐seeking adolescents^70^ highlighted that family history may influence developmental factors which in turn would lead to dietary restraint (Figure 5D).

#### 3.4.5 Bulimia

**FIGURE 6:**
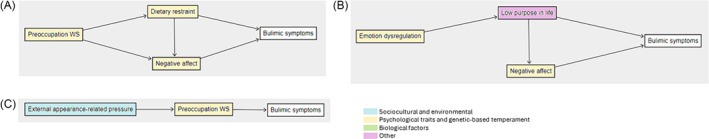
Pathways to bulimic symptoms assessed in (A) adults not seeking obesity treatment (*n* = 1 study), (B) adults seeking obesity treatment (*n* = 1 study), and (C) adolescents not seeking obesity treatment (*n* = 1 study). *Note*: Individual pathways from multiple studies have been collated to form figures; statistical significance was not considered, and complete represented model with all variables has not been tested together. Preoccupation WS = preoccupation with weight and shape.

Three studies looked at bulimic symptoms or behaviors. One study hypothesized that dietary restraint and negative affect mediate the pathways between preoccupation with weight and shape and bulimic symptoms in non‐treatment‐seeking adults (Figure 6A).^23^ In treatment‐seeking adults with obesity, one study proposed that emotion regulation leads to bulimic symptoms through low purpose in life and negative affect (Figure 6B).^53^ A single study of a non‐treatment‐seeking adolescent cohort^75^ examined whether external appearance‐related pressure resulted in bulimic symptoms through preoccupation with weight and shape (Figure 6C).

#### 3.4.6 Longitudinal studies

Three studies examined mediation using longitudinal data, and the results are summarized in Table S3. Anderson et al. (2022) examined whether the relationship between “feeling fat” and an objective binge eating episode was mediated by disgust, guilt, or shame among adults who self‐identify as living with a higher weight.^36^ Mediation did not occur in any of the longitudinal (within subjects) univariate or multivariate models (*p* > 0.05). Rayner, Schniering, Rapee, and Hutchinson (2013) examined whether the relationship between perceived friend influence and the two outcomes, bulimic behaviors and dieting, were mediated by body dissatisfaction in an Australian sample of female high school students.^75^ They did not find body dissatisfaction significantly mediated either outcome (*p* > 0.05). Wardle, Waller, and Rapoport (2001) examined whether the relationship between body dissatisfaction and binge eating score was mediated by either depression or restraint.^64^ They found depression partially mediated the effect of body dissatisfaction on BES; however they did not investigate the mediation of restraint on BES score as it was not associated with the outcome. A follow up interaction test produced weak evidence that as depression decreased and restraint increased, BES symptoms decreased (*p* = 0.06).

### 3.5 Stakeholder review of identified models

Consultation with stakeholders with lived experience of EDs and obesity revealed general agreement with model constructs and pathways identified. However, there was emphasis on key factors that were missing. These included parental feeding practices, childhood trauma, genetic factors, health‐care provider attitudes, and weight talk, as well as the lack of research on adolescents.

## 4 DISCUSSION

This present study is the first to review conceptual models of ED development that have been evaluated in people with overweight and obesity. Forty‐six models proposed over 100 different pathways, highlighting the complexity of the development of EDs and related behaviors in people with overweight and obesity. Of the 18 constructs observed across the ED models, negative affect, preoccupation with weight and shape, and weight stigma were the most common to form potential pathways, and these appeared to account for the most variance in models in studies where these data were reported. Similar pathways were proposed across ED outcomes; however, binge eating models reported additional constructs such as food insecurity and insomnia symptoms. This is likely because of more than half the identified studies examining binge eating, with fewer studies assessing global ED psychopathology, loss of control eating, dietary restraint, and bulimia. No studies were identified assessing for pathways to the development of anorexia nervosa, fasting, laxative abuse, or excessive exercise in people with overweight and obesity. Most models focused on adults and predominantly White females from developed countries. Moreover, studies more commonly included obesity treatment‐seeking populations than community samples and only one study examined pathways by sex. Further research is required for adolescents overall, males, community samples representative of the population (in context of ethnicity and socio‐economic status, and obesity treatment‐seeking status) and the spectrum of EDs for adults beyond binge eating, especially atypical anorexia nervosa.

Negative affect and preoccupation with weight and shape were the most common constructs, forming one‐third of the variables identified. They were theorized to mediate the pathways between various constructs and ED outcomes in 80% of models. A previous review of 623 studies, with no restriction on weight status, also found these to be common constructs within models of ED development.^21^ This aligns with research that suggests negative affect and preoccupation with weight and shape are strong risk factors for the spectrum of EDs.^6^, ^28^ These findings are also consistent with the Dual‐Pathway Model for bulimia which includes negative affect and preoccupation with weight and shape as central mediators in pathways to bulimia symptomology.^22^, ^77^ Dietary restraint, emotion regulation, external appearance‐related pressure, self‐esteem deficits, interpersonal issues, developmental factors, and body ideal internalization were also constructs identified both in this study and previous research on ED models.^21^ While these constructs were observed infrequently in our study across different subgroups, few studies reported the variance attributable to these factors, with these data only available for self‐esteem (27–54%) and emotional dysregulation (33–36%) in three studies each. It is possible that these constructs play an important role in ED development and should be further evaluated in people with overweight and obesity of different ages, backgrounds, and treatment‐seeking statuses.

Weight stigma was the third most frequently proposed construct to lead to EDs and related behaviors, reported in 10 of 46 studies. Weight stigma refers to people with overweight and obesity experiencing negative stereotypes, discriminative treatment, devaluation, and prejudice because of their weight.^78^ Evidence suggests that weight stigma is strongly associated with disordered eating.^79^ Indeed, this review found that weight stigma was proposed to precede nine different constructs leading to ED outcomes. These include emotion regulation, self‐esteem deficits, preoccupation with weight and shape, and negative affect. The potential significance of weight stigma in a number of pathways identified in this study aligns with a recent survey of clinicians, researchers, and consumers identifying weight stigma as a key predictor of ED risk for people with overweight and obesity.^14^ However, previous reviews with a greater focus on people with lower weight do not report on weight stigma as a risk factor or as part of models for EDs development.^12^, ^21^, ^28^ Moreover, most current ED screening tools do not assess for experience of weight stigma.^80^ This may, in and of itself, be a reflection of weight stigma in research and health care. Given that weight stigma is particularly pervasive, there is a need to increase representation of this population in research on understanding the development of EDs.

Risk factors with the themes of experiential avoidance, interoceptive deficits, low control over eating, hunger, food insecurity, and parental psychopathology are not frequently reported in previous reviews of ED models assessing predominately lower weight populations.^21^ Potential predictive variables that were observed infrequently and did not match any themes included adult ADHD, insomnia symptom severity, low self‐monitoring, low purpose in life, low perceived control, PTSD symptoms, and executive function difficulties. While these factors are not commonly assessed as part of larger conceptual models, varied research exists on these variables as individual risk factors for EDs.^81^, ^82^, ^83^, ^84^, ^85^ Nevertheless, numerous ED risk factors were not considered in the models identified in this review, including some that are well‐established in people with lower weight, such as childhood abuse and trauma,^86^ and those which are strongly believed to be influential by clinicians, researchers, and people with lived experience in the field of EDs and obesity (e.g., environmental context such as social media use and parental weight talk).^14^, ^87^, ^88^ A comprehensive model encompassing psychological, social, and biological pathways to ED development is important to illustrate the complex aetiology of the disease and variations in disease manifestations between individuals. This is important for not only screening and treatment but also for reducing stigma which has been demonstrated through explaining the aetiology of EDs.^89^

## 5 CONCLUSION

Findings from this review highlight both the complex interplay of potential pathways to ED development in people with overweight and obesity, and the lack of research available in adolescents and on disorders other than binge eating. Of the models identified, negative affect, preoccupation with weight, and shape and weight stigma were the most common themes forming pathways to the development of different EDs and related disordered eating behaviors. This study highlights the need to establish evidence‐based development pathways specific to different EDs in people with overweight and obesity to improve prevention, identification, and treatment in this at‐risk population.

### 5.1 Strengths and limitations

This review is the first to recognize the need to identify pathways to ED development that are specific to people with overweight and obesity. A comprehensive systematic search of the literature was conducted to summarize available evidence on ED development models that have been evaluated in people with overweight and obesity. Study review design and results were also reviewed by people with lived experience of both overweight and obesity and EDs with key contributions including discussion points and highlighting gaps in current literature. A wide range of risk factors and pathways were thematically described for different eating outcomes and population subgroups. There were several limitations. Most included studies were cross‐sectional. Many have^31^, ^32^ argued that cross‐sectional studies provide biased information about the role of risk factors over time for two reasons: (1) they cannot control for initial levels of risk factors, and (2) causal changes occur over time. Because of this, models of causal pathways may be confounded if they do not take time into account and instead allow cause to simultaneously have an effect. Where possible, we have reported *R* ^2^ (proportion of explained variance), however, models included in our review vary widely in their complexity. In many cases, we would expect more complex models that include many parameters to account for greater amounts of variance. At a pragmatic level, adjusted *R* ^2^ and information criterion measures of model fit that penalize for the amount of parameters included (e.g., BIC), and external model validation metrics were reported heterogeneously or not at all. We did not consider whether the pathways identified were statistically significant. Reliable interpretation of significance would require critical analysis of the statistical methods used in each included study, and assessment of effect sizes in addition to confidence intervals was beyond the scope of this review. The direction of effect of pathways described is also based on what has been proposed by included studies and may not represent true directions or potential bi‐directional pathways.

## AUTHOR CONTRIBUTIONS

Rabia Khalid, Natalie B. Lister, Susan J. Paxton, Sarah Maguire, Sol Libesman, Anna L. Seidler, Louise A. Baur, and Hiba Jebeile contributed to the study protocol. Rabia Khalid conducted the search, screening, and stakeholder consultation. Rabia Khalid, Sol Libesman, and Hiba Jebeile conducted the data extraction and results synthesis. All authors contributed to the interpretation of data. Rabia Khalid drafted the manuscript. All authors critically revised the article for important intellectual content and approved the final version to be published.

## CONFLICT OF INTEREST STATEMENT

The authors declare no conflict of interest.

## Supporting information


**Table S1:** Search strategies.
**Table S2**: Characteristics of included studies.
**Table S3**: Mediation results of pathways in longitudinal studies.
**Figure S1**: PRISMA Flow Diagram of study selection.
