# Supplementary material for: Potential pathways to the onset and development of eating disorders in people with overweight and obesity: A scoping review
Source: Obes Rev. 2024 Oct 4;26(1):e13840. doi: 10.1111/obr.13840 (PMC11611440; doi:10.1111/obr.13840)
Supplement: Supplementary file 1 — Table S1: Search strategies. Table S2: Characteristics of included studies. Table S3: Mediation results of pathways in longitudinal studies. Figure S1: PRISMA Flow Diagram of study selection. [file OBR-26-e13840-s001.pdf]

Conceptual frameworks for the onset and development of eating disorders in people with higher weight: a scoping review – supplementary material

Authors: Rabia Khalid[1,2], Natalie B Lister[1,2], Susan J Paxton[3], Sarah Maguire[4], Sol Libesman[5], Anna L Seidler[5], Kelly Cooper[6], Fiona Quigley[7], Jacqlyn Yourell[8], Louise A Baur[1,9], Hiba Jebeile[1,2]

[1] The University of Sydney, Children's Hospital Westmead Clinical School, Westmead, New South Wales, Australia.

[2] Charles Perkins Centre, The University of Sydney, New South Wales, Australia.

[3] School of Psychology and Public Health, La Trobe University, Melbourne, Victoria, Australia.

[4] InsideOut Institute for Eating Disorders, Charles Perkins Centre, The University of Sydney, Sydney, New South Wales, Australia.

[5] National Health and Medical Research Council Clinical Trials Centre, The University of Sydney, Sydney, New South Wales, Australia.

[6] Weight Issues Network, New South Wales, Australia

[7] Institute of Nursing and Health Research, Ulster University, Belfast, Northern Ireland.

[8] Department of Family, Youth and Community Sciences, University of Florida College of Agricultural and Life Sciences, Gainesville, Florida, USA.

[9] Weight Management Services, The Children's Hospital at Westmead, Westmead, New South Wales, Australia.

Corresponding author: Rabia Khalid, The University of Sydney, Children's Hospital Westmead Clinical School, Westmead, Australia
Email: rabia.khalid@sydney.edu.au

Table S1: Search strategies:

**Ovid MEDLINE(R) ALL <1946 to July 25, 2022>**		
1	exp Obesity/	248282
2	exp Overweight/	259188
3	obes*.tw.	350891
4	overweight.tw.	81586
5	high* weight.tw.	3799
6	high* BMI.tw.	13781
7	high* body mass index.tw.	9922
8	1 or 2 or 3 or 4 or 5 or 6 or 7	438403
9	exp Models, Psychological/	47102
10	(pathway* adj4 model*).tw.	11320
11	(concept* adj4 model*).tw.	20444
12	exp Models, Statistical/	445928
13	framework.tw.	328145
14	mediat*.tw.	1514153
15	(predict* adj4 (eating disorder* or binge or bulimi* or anorexi*)).tw.	1255
16	(risk adj4 (eating disorder* or binge or bulimi* or anorexi*)).tw.	2365
17	(protect* adj4 (eating disorder* or binge or bulimi* or anorexi*)).tw.	170
18	9 or 10 or 11 or 12 or 13 or 14 or 15 or 16 or 17	2321261
19	"feeding and eating disorders"/ or anorexia nervosa/ or binge-eating disorder/ or bulimia nervosa/ or "feeding and eating disorders of childhood"/ or orthorexia nervosa/	33060
20	Bulimia/	5769
21	((disorder* or binge) adj4 eat*).tw.	27226
22	19 or 20 or 21	44302
23	8 and 18 and 22	1132
24	exp animals/ not humans.sh.	5039593
25	23 not 24	1093
26	limit 25 to english	1042
**Embase Classic+Embase <1947 to 2022 July 25>**		
1	exp obese patient/ or exp obesity/	620006
2	overweight.tw.	122577
3	obes*.tw.	524591
4	high* weight.tw.	5451
5	high* BMI.tw.	26396
6	high* body mass index.tw.	13548
7	1 or 2 or 3 or 4 or 5 or 6	757321
8	exp psychological model/	30397
9	(pathway* adj4 model*).tw.	14401
10	(concept* adj4 model*).tw.	23656
11	framework.tw.	358785
12	mediat*.tw.	1865986
13	exp statistical model/	650158
14	(predict* adj4 (eating disorder* or binge or bulimi* or anorexi*)).tw.	1495
15	(risk adj4 (eating disorder* or binge or bulimi* or anorexi*)).tw.	2991
16	(protect* adj4 (eating disorder* or binge or bulimi* or anorexi*)).tw.	216
17	8 or 9 or 10 or 11 or 12 or 13 or 14 or 15 or 16	2890656
18	exp eating disorder/ or exp anorexia nervosa/ or exp binge eating disorder/ or exp bulimia/ or exp orthorexia/ or exp purging disorder/	61202
19	((disorder* or binge) adj4 eat*).tw.	35028
20	18 or 19	66722
21	7 and 17 and 20	1791
22	limit 21 to "humans only (removes records about animals)"	1711

23 limit 22 to english language 1633

APA PsycInfo <1806 to July Week 3 2022>

1 exp Obesity/ 27277
2 exp Overweight/ 28868
3 obes*.tw. 45494
4 overweight.tw. 16870
5 high* weight.tw. 666
6 high* BMI.tw. 1868
7 high* body mass index.tw. 1031
8 1 or 2 or 3 or 4 or 5 or 6 or 7 52073
9 "Models, Psychological".mh. 28296
10 (pathway* adj4 model*).tw. 2441
11 (concept* adj4 model*).tw. 22748
12 exp statistical analysis/ 140253
13 framework.tw. 205430
14 mediat*.tw. 230623
15 (predict* adj4 (eating disorder* or binge or bulimi* or anorexi*)).tw. 1499
16 (risk adj4 (eating disorder* or binge or bulimi* or anorexi*)).tw. 2468
17 (protect* adj4 (eating disorder* or binge or bulimi* or anorexi*)).tw. 144
18 9 or 10 or 11 or 12 or 13 or 14 or 15 or 16 or 17 598361
19 eating disorders/ or exp anorexia nervosa/ or exp binge eating disorder/ or exp bulimia/ or exp "purging (eating disorders)"/ or exp binge eating/ 33618
20 ((disorder* or binge) adj4 eat*).tw. 32779
21 19 or 20 41880
22 8 and 18 and 21 908
23 exp animals/ not humans.sh. 368709
24 22 not 23 880
25 limit 24 to english 825

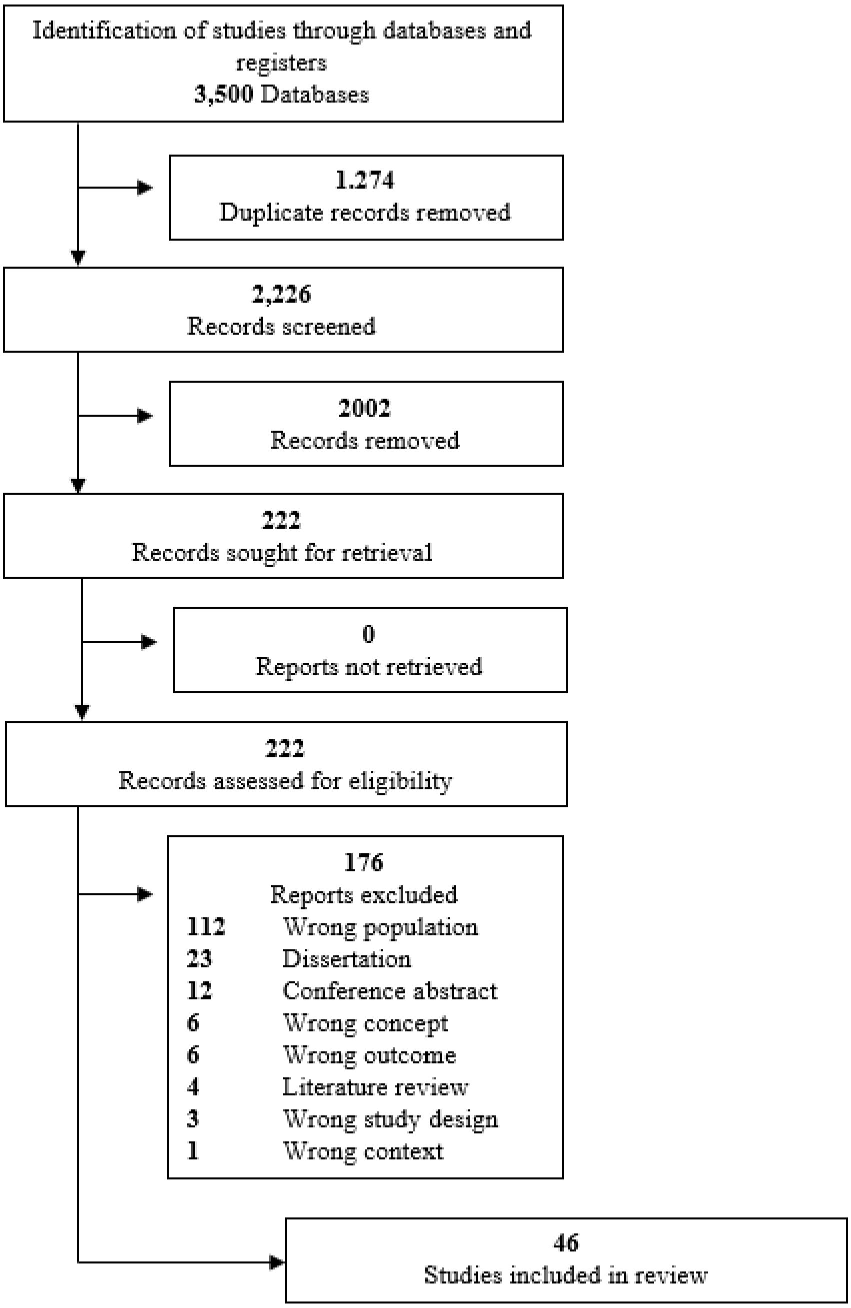

Figure S1: PRISMA Flow Diagram of study selection

Table S2: Characteristics of included studies:

Study ID (author, year)	Country	Study design	Population age group	Seeking obesity treatment	ED outcome (measurement scale)	Sample size	Mean age: [years (SD)]	Sex (% female)	Mean BMI [kg/m2 (SD)]	Main ethnicity (% total)	Population/ Recruitment setting
Elarchi S 2021[1]	France	Cross-sectional	Adults	Yes	Binge eating (BES)	343	43.1 (11.2)	76.6%	45.4 (7.7)	*Not reported*	Adult bariatric surgery candidates
Almenara C.A 2017[2]	Canada	Cross-sectional	Adults	Yes	Dietary restraint (EDE-Q6)	111	40.9 (10.20)	100%	*Not reported*	*Not reported*	Females attending eating- and weight-related problems private outpatient clinic
Aloi M 2020[3]	Italy	Cross-sectional	Adults	No	Binge eating severity (BES)	46	40.6 (12.7)	87%	38.8 (7.1)	*Not reported*	Adults with binge-eating disorder attending outpatient eating disorder clinic
Anderson L 2022[4]	USA	Longitudinal	Adults	No	Current ED, Life-time ED, Objective binges (SCID-I/P)	50	43.0 (11.9)	84%	40.3 (8.5)	White (76%)	Community sample of adults with self-reported higher weight
Baldofski S 2016[5]	Germany	Cross-sectional	Adults	Yes	Global eating pathology (EDE-Q; EAH; DEBQ)	240	45.08 (10.43)	69%	48.62 (7.48)	*Not reported*	Bariatric surgery candidates
Cella S 2019[6]	Italy	Cross-sectional	Adults	Yes	Binge eating behaviour (BES)	61	35.34 (10.37)	71%	41.03 (5.55)	Caucasian (71%)	Bariatric surgery candidates
Cheung V 2022[7]	USA	Cross-sectional	Adults	Yes	Binge eating behaviour (BES)	369	42.67 (11.15)	80%	*Not reported*	White (94.9%)	Bariatric surgery candidates
Clarke M 2000[8]	USA	Case-control	Adults	Yes	Binge eating (QEWP-5)	43	42.59 (8.29)	100%	40 (6.84)	Caucasian (97.6%)	Females diagnosed with binge eating disorder attending hospital-based weight

Study ID (author, year)	Country	Study design	Population age group	Seeking obesity treatment	ED outcome (measurement scale)	Sample size	Mean age: [years (SD)]	Sex (% female)	Mean BMI [kg/m2 (SD)]	Main ethnicity (% total)	Population/ Recruitment setting
											management program
Coffino J 2016[9]	USA	Cross-sectional	Adults	No	Binge eating severity (BES)	173	19.00 (2.04)	45%	28.08 (3.28)	White (47.2%)	Undergraduate university students
Conti C 2019[10]	Italy	Cross-sectional	Adults	Yes	Binge eating severity (BES)	361	45.74 (14.24)	70%	35.28 (6.92)	*Not reported*	Adults referred to obesity centre
Cox S 2018[11]	USA	Cross-sectional	Adults	Yes	Binge eating (BSM-IV-TR; BES)	119	46.68 (12.01)	85%	50.77 (10.01)	White (97%)	Bariatric surgery candidates
Darling K 2020[12]	USA	Cross-sectional	Adolescents	Yes	Disordered eating behaviour (EDE-Q)	170	14.81 (1.82)	62.4%	Mean BMI % of the 95th percentile (CDC charts): 142.24 (32.89)	White (48.2%)	Adolescents in hospital-based weight management program
Decauwe´ V 2006[13]	Belgium	Cross-sectional	Adolescents	Yes	Dietary restraint (ChEDE)	272	12.7 (1.8)	Not reported	31.2 (5.3)	*Not reported*	Children and adolescents seeking obesity treatment in inpatient or outpatient setting
Decauwe´ V 2005[14]	Belgium	Cross-sectional	Adolescents	Yes	Binge eating (ChEDE)	196	12.73 (1.75)	60.2%	Mean overweight of 172.69% (27.09)	*Not reported*	Children and adolescents seeking obesity treatment in inpatient or outpatient setting
Duarte C 2015[15]	UK	Cross-sectional	Adults	Yes	Eating disinhibition (TFQ)	2236	41.71 (12.34)	100%	31.62 (6.10)	*Not reported*	Females attending community-based weight

Study ID (author, year)	Country	Study design	Population age group	Seeking obesity treatment	ED outcome (measurement scale)	Sample size	Mean age: [years (SD)]	Sex (% female)	Mean BMI [kg/m2 (SD)]	Main ethnicity (% total)	Population/ Recruitment setting
											management programme
Durso L 2015[16]	USA	Cross-sectional	Adults	No	Bulimia; Drive for thinness; Frequency of binge eating (EDDS; EES; EDI)	228 (with overweight; 486 total)	39.06 (11.45)	81.7%*	37.71 (13.20)	Caucasian (79.5%)*	Community sample of adults (results reported separately for those with overweight/obesity)
Fox J 2016[17]	England	Cross-sectional	Adults	Yes	Binge eating (BES)	51	47 (11.47)	70.5%	43.5 (6.12)	*Not reported*	Adults (pre-treatment) enrolled in weight management services
Gagnon-Girouard M 2009[18]	Canada	Cross-sectional	Adults	No	Disinhibition; Binge eating (TFQ; BES)	153	42.4 (5.6)	100%	30.46 (3.03)	*Not reported*	Pre-menopausal women with stable higher weight
Gagnon-Girouard M 2010[19]	Canada	Cross-sectional	Adults	Yes	Disordered eating behaviour (EDE-Q)	22	42.3 (5.5) for total sample (not reported for sample of interest)	100%	30.09 (2.80)	*Not reported*	Females participating in a healthy lifestyle promotion intervention
Gerke C 2013[20]	USA	Cross-sectional	Adolescents	Yes	Global eating pathology (ChEDE)	92	13.92 (22.05)	67%	Mean BMI z-score: 2.50 (0.25)	African American (78%)	Adolescents enrolled in weight management program

Study ID (author, year)	Country	Study design	Population age group	Seeking obesity treatment	ED outcome (measurement scale)	Sample size	Mean age: [years (SD)]	Sex (% female)	Mean BMI [kg/m2 (SD)]	Main ethnicity (% total)	Population/ Recruitment setting
Goosens L 2010[21]	Belgium	Cross-sectional	Adolescents	Mixed	Loss of control eating (ChEDE)	350	13.30 (2.17)	59.7%	31.27 (5.6); 168.78% (27.21) adjusted BMI based on CDC BMI z-scores	*Not reported*	Children and adolescents with overweight and obesity (either seeking or not seeking treatment)
Goosens L 2010[22]	Belgium	Cross-sectional	Adolescents	Mixed	Loss of control eating (ChEDE)	188	13.76 (2.33)	60.1%	31.32 (5.97); 167.18% (28.0) adjusted BMI based on CDC BMI z-scores	*Not reported*	Children and adolescents with overweight and obesity (either seeking or not seeking treatment)
Hecht L 2020[23]	USA	Cross-sectional	Adults	Yes	Global eating pathology (EDE-Q)	88	43.31 (11.55)	85%	50.63 (10.85)	African American (53.5%)	Adult bariatric surgery candidates
Iannaccone M 2016[24]	Italy	Cross-sectional	Adolescents	No	Eating disorder risk (EDI-3)	111	15.5 (1.5)	38.7%	29.69 (3.43)	*Not reported*	Public high school students
Jankauskiene R 2020[25]	Lithuania	Cross-sectional	Adults	No	Disordered eating behaviour (EDE-Q6)	193	20.4 (3.1)	0%	*Not reported*	*Not reported*	Lithuanian college and university students

Study ID (author, year)	Country	Study design	Population age group	Seeking obesity treatment	ED outcome (measurement scale)	Sample size	Mean age: [years (SD)]	Sex (% female)	Mean BMI [kg/m2 (SD)]	Main ethnicity (% total)	Population/ Recruitment setting
Kelly N 2020[26]	USA	Cross-sectional	Adults	No	Loss of control eating frequency (EDE-Q)	1109*	24.1 (3.6)*	0%	25.4 (6.2)*	White/Caucasian (28.4%)*	Community sample of males
Kenny T 2017[27]	Canada	Cross-sectional	Adults	Yes	Binge frequency (EDE-Q6)	68	40.44 (11.63)	92.6%	37.57 (9.58)	White (97.1%)	Adults enrolled in binge eating disorder treatment trial
Lo Coco G 2016[28]	Italy	Cross-sectional	Adults	No	Binge eating (BES)	186	45.1 (14.5)	66.7%	38.3 (6.7)	*Not reported*	Adults referred to eating and weight disorders clinic
Marco J 2021[29]	Spain	Cross-sectional	Adults	Yes	Bulimia nervosa or binge eating symptoms (BITE)	122	47 (9.89)	64.8	44.41 (6.03)	Caucasian (100%)	Bariatric surgery hospital-waitlist patients
Munir M 2020[30]	Pakistan	Cross-sectional	Adolescents	No	Eating withdrawal; Overeating (DEBS)	200	17.37 (1.50)	100%	29.81 (2.92)	*Not reported*	Adolescent female university and college students
Palmeira L 2017[31]	Portugal	Cross-sectional	Adults	Yes	Binge eating (BES)	124	41.14 (8.72)	100%	34.44 (5.51)	*Not reported*	Females enrolled in nutritional weight loss programs
Palmeira M 2018[32]	Portugal	Cross-sectional	Adults	Yes	Uncontrolled eating (TFEQ)	331	44.11 (10.90)	100%	32.11 (4.60)	*Not reported*	Females enrolled in nutritional weight loss programs

Study ID (author, year)	Country	Study design	Population age group	Seeking obesity treatment	ED outcome (measurement scale)	Sample size	Mean age: [years (SD)]	Sex (% female)	Mean BMI [kg/m2 (SD)]	Main ethnicity (% total)	Population/ Recruitment setting
Palmeira M 2019[33]	Portugal	Cross-sectional	Adults	Yes	Eating psychopathology severity (EDEQ)	203	40.08 (11.74)	49.8%	31.17 (5.43)	*Not reported*	Adults enrolled in nutritional weight loss programs
Peterson J 2021[34]	USA	Cross-sectional	Adults	Yes	Cognitive restraint (TFEQ)	79	38.56 (12.12)	92.4%	33.78 (5.69)	White (92.4%)	Adults interested in online nutrition and physical activity program
Rania M 2021[35]	Italy	Case-control	Adults	Yes	Binge eating severity (BES)	66	47.6 (10.6)	53.7%	39.5 (11.3)	*Not reported*	Adults seeking specialist treatment for binge eating disorder or obesity in outpatient setting
Rayner K 2012[36]	Australia	Longitudinal	Adolescents	No	Bulimic behaviour; Dieting (DEBQ; EDI)	1094 at baseline (total sample of which 15-19% had overweight and 6-7% had obesity across timepoints)	12.3 (0.52)*	100%	*Not reported*	*Not reported*	Adolescent females from urban Australian high schools
Rice A 2022[37]	USA	Cross-sectional	Adolescents	Yes	Disordered eating attitudes (EDE)	149	(14.4 (1.6)	55%	Mean BMI z-score (SD): 1.9 (0.4)	Non-Hispanic White (37.3%)	Adolescent military dependents enrolled in adult obesity and binge eating disorder

Study ID (author, year)	Country	Study design	Population age group	Seeking obesity treatment	ED outcome (measurement scale)	Sample size	Mean age: [years (SD)]	Sex (% female)	Mean BMI [kg/m2 (SD)]	Main ethnicity (% total)	Population/ Recruitment setting
											prevention intervention
Shakory S 2015[38]	Canada	Cross-sectional	Adults	Yes	Binge eating (BES)	1388	44.69 (10.59)	79.3%	49.00 (8.67)	White (84.8%)	Bariatric surgery candidates
Shaumberg K 2016[39]	USA	Cross-sectional	Adults	Yes	Disinhibition (TFQ)	107	53.3 (9.7)	88.8%	36.6 (5.1)	White (56.1%)	Adults enrolled in a behavioural weight loss intervention
Smith K 2018[40]	USA	Cross-sectional	Adults	No	Binge eating (Other - study specific)	50	43.0 (11.9)	82	40.3 (8.5)	White (76%)	Community sample of adults
Soulliard Z 2020[41]	USA	Cross-sectional	Adults	Yes	Binge eating (BES)	708	42.71 (11.00)	79.40%	48.92 (7.81)	White (95.3%)	Adult bariatric surgery candidates
Wardle J 2001[42]	UK	Longitudinal	Adults	Yes	Binge eating (BES)	89	47 (11)	100%	36 (6)	White (72%)	Females participating in weight and lifestyle intervention
Wellman J 2019[43]	USA	Cross-sectional	Adults	No	Binge eating (BES)	538	21.81 (5.46)	75.10%	30.85 (5.43)	Hispanic/Latino (54.6%)	College students

Study ID (author, year)	Country	Study design	Population age group	Seeking obesity treatment	ED outcome (measurement scale)	Sample size	Mean age: [years (SD)]	Sex (% female)	Mean BMI [kg/m2 (SD)]	Main ethnicity (% total)	Population/ Recruitment setting
Welsh D 2016[44]	Australia	Cross-sectional	Adults	No	Bulimic symptoms; Dietary restraint (BULIT; DRES; PANAS-SF-NA)	260*; 145 with overweight or obesity	28.19 (9.38)*	92.30%	31.54 (6.22) for sample with overweight	Caucasian (88%)	Self-identified binge eaters completing online international survey
Wooldridge J 2021[45]	USA	Cross-sectional	Adults	Yes	Binge eating symptoms (BES)	89	55.97 (10.86)	22.50%	37.5 (7.3)	White (68.5%)	Military veterans enrolled in problematic eating intervention
Zickgraf H 2019[46]	USA	Cross-sectional	Adults	Yes	Binge eating syndrome (BES)	240	41.09 (11.84)	75.00%	48.33 (7.56)	White (71.3%)	Adult bariatric surgery candidates

Note: *Results reported for total study sample when data for participants with overweight and obesity was not reported separately. BES = Binge Eating Scale; BITE = Bulimic Investigatory Test Edinburgh; BSM-IV-TR = Diagnostic and Statistical Manual of Mental Disorders Text Revision Fourth Edition; BULIT = Bulimia Test; ChEDE = Child Eating Disorder Examination; DEBQ = Dutch Eating Behavior Questionnaire; DEBS = Disordered Eating Behavior Scale; DRES = Dutch Restrained Eating Scale; ED = eating disorder; EDDS = Eating Disorder Diagnostic Scale; EDE = Eating Disorder Examination; EDE-Q = Eating Disorder Examination Questionnaire; EDI = Eating Disorder Inventory; PANAS = Positive and Negative Affect Scale; QEWP-5 = Questionnaire on Eating and Weight Patterns 5.0; SCID-I/P = Structured clinical interview for DSM-IV axis I disorders, patient edition: eating disorder module; TFEQ = Three Factor Eating Questionnaire; TFQ = Three Factor Questionnaire

Table S3: Mediation results of pathways in longitudinal studies

Trial	Predictor	Mediator(s)	Outcome	Effect	p value
Anderson L 2022[4]	Feeling fat	disgust	Objective binge episode	Indirect effect = 0.005 (0.007)	p=0.435
		guilt		Indirect effect = -0.004 (0.009)	p=0.602
		shame		Indirect effect = -0.003 (0.12)	p =0.602
		Digust + guilt+ shame		Adjusted indirect effect digust 0.01 (0.011)<br><br>Adjusted indirect effect guilt - 0.007 (0.012)<br><br>Adjusted indirect effect shame -0.005 (0.018)	p=0.348<br><br>p=0 .566<br><br>p=.0764
Rayner K 2013[36]	Perceived friend influence (Peer influence to be Thin and Diet Scale, Friends Concern with Thinness and Dieting Scale, Friends as a Source of Influence Scale)	Body dissatisfaction (Eating Disorder Inventory – Body dissatisfaction, Body Attitudes Questionnaire)	Bulimic Behaviours (Dutch Eating Behaviour Questionnaire Restrain subscale parcel indicator 1)	Final model RMSEA = 0.029, Total effect: β=-0.002 Friend influence was not a significant mediator (effect not reported)	Total effect P=0.079<br><br>Mediation effect p>0.05
			Dieting (Dutch Eating Behaviour Questionnaire Restrain subscale parcel indicator 2)	Final model RMSEA = 0.029, Total effect: β=-0.002 Friend influence was not a significant mediator (effect not reported)	Total effect p=0.247<br><br>Mediation effect p>0.05
Wardle J 2001[42]	Body dissatisfaction	Depression reduction	Binge-eating behaviour was assessed with the Binge Eating Scale (BES)	Adjusted R^2 =0.29. Unique BES variance explained by body dissatisfaction reduces from 6% to 1% when including depression as a mediator.	Total effect p=0.007<br><br>Association between depression and BES adjusting for body dissatisfaction, weight lost and treatment p<0.01

		restraint	Binge-eating behaviour was assessed with the Binge Eating Scale (BES)	Restraint was not significantly associated with BES and the main effect mediation was not investigated.	Not reported

References

1. El Archi S, Brunault P, De Luca A, et al. Do Emotion Dysregulation, Alexithymia and Personality Dimensions Explain the Association Between Attention-Deficit/Hyperactivity Disorder and Binge Eating Among Bariatric Surgery Candidates? *Frontiers in psychology*. 2021;12:745857. doi:https://dx.doi.org/10.3389/fpsyg.2021.745857
2. Almenara CA, Aime A, Maiano C, et al. Weight stigmatization and disordered eating in obese women: The mediating effects of self-esteem and fear of negative appearance evaluation. Eating Disorders 3260. *European Review of Applied Psychology / Revue Europeenne de Psychologie Appliquee*. 2017;67(3):155-162. Revue de Psychologie Appliquee. doi:https://dx.doi.org/10.1016/j.erap.2017.02.004
3. Aloi M, Rania M, Carbone EA, et al. The role of self-monitoring metacognition sub-function and negative urgency related to binge severity. *European eating disorders review : the journal of the Eating Disorders Association*. 2020;28(5):580-586. doi:https://dx.doi.org/10.1002/erv.2742
4. Anderson LM, Hall LMJ, Crosby RD, et al. "Feeling fat," disgust, guilt, and shame: Preliminary evaluation of a mediation model of binge-eating in adults with higher-weight bodies. *Body image*. 2022;42:32-42. doi:https://dx.doi.org/10.1016/j.bodyim.2022.05.008
5. Baldofski S, Rudolph A, Tigges W, et al. Weight bias internalization, emotion dysregulation, and non-normative eating behaviors in prebariatric patients. *The International journal of eating disorders*. 2016;49(2):180-5. doi:https://dx.doi.org/10.1002/eat.22484
6. Cella S, Cipriano A, Giardiello C, Cotrufo P. Relationships Between Self-Esteem, Interoceptive Awareness, Impulse Regulation, and Binge Eating. Path Analysis in Bariatric Surgery Candidates. *Clinical neuropsychiatry*. 2019;16(5-6):213-220. doi:https://dx.doi.org/10.36131/clinicalnpsych2019050604
7. Cheung V, Aylward L, Tabone L, Szoka N, Abunnaja S, Cox S. Hunger mediates the relationship between food insecurity and binge eating among bariatric surgery candidates. *Surgery for obesity and related diseases : official journal of the American Society for Bariatric Surgery*. 2022;18(4):530-537. doi:https://dx.doi.org/10.1016/j.soard.2021.12.009
8. Clark MM, Forsyth LH, Lloyd-Richardson EE, King TK. Eating self-efficacy and binge eating disorder in obese women. Eating Disorders 3260. *Journal of Applied Biobehavioral Research*. 2000;5(2):154-161. doi:https://dx.doi.org/10.1111/j.1751-9861.2000.tb00071.x
9. Coffino JA, Orloff NC, Hormes JM. Dietary Restraint Partially Mediates the Relationship between Impulsivity and Binge Eating Only in Lean Individuals: The Importance of Accounting for Body Mass in Studies of Restraint. *Frontiers in psychology*. 2016;7:1499.
10. Conti C, Di Francesco G, Lanzara R, et al. Alexithymia and binge eating in obese outpatients who are starting a weight-loss program: A structural equation analysis. *European eating disorders review : the journal of the Eating Disorders Association*. 2019;27(6):628-640. doi:https://dx.doi.org/10.1002/erv.2696
11. Cox S, Brode C. Predictors of Binge Eating among Bariatric Surgery Candidates: Disinhibition as a Mediator of the Relationship Between Depressive Symptoms and Binge Eating. *Obesity surgery*. 2018;28(7):1990-1996. doi:https://dx.doi.org/10.1007/s11695-018-3129-8
12. Darling KE, Ranzenhofer LM, Hadley W, Villalta D, Kasper V, Jelalian E. Negative childhood experiences and disordered eating in adolescents in a weight management program: The role of depressive symptoms. *Eating behaviors*. 2020;38:101402. doi:https://dx.doi.org/10.1016/j.eatbeh.2020.101402
13. Decaluwe V, Braet C, Moens E, Van Vlierberghe L. The association of parental characteristics and psychological problems in obese youngsters. *International journal of obesity (2005)*. 2006;30(12):1766-74.
14. Decaluwe V, Braet C. The cognitive behavioural model for eating disorders: a direct evaluation in children and adolescents with obesity. *Eating behaviors*. 2005;6(3):211-20.
15. Duarte C, Matos M, Gail C, Morris L, Stubbs RJ, Gilbert P. The impact of shame, self-criticism and social rank on eating psychopathology in overweight and obese members of a commercial weight management programme. *Obesity Facts*. 2015;8(SUPPL. 1):182. 22nd Congress of the European Congress on Obesity, ECO 2015. Prague Czechia.

(var.pagings). doi:https://dx.doi.org/10.1159/000382140

16. Durso LE, Latner JD, Hayashi K. Perceived discrimination is associated with binge eating in a community sample of non-overweight, overweight, and obese adults. *Obesity facts*. 2012;5(6):869-80. doi:https://dx.doi.org/10.1159/000345931
17. Fox JRE, Msetfi RM, Johnson RS, Haigh E. The Perception of Threat from Emotions in Predicting Binge Eating Behaviours in People Who Are Obese and Seeking Treatment for Their Weight. *Clinical psychology & psychotherapy*. 2016;23(5):452-459. doi:https://dx.doi.org/10.1002/cpp.1972
18. Gagnon-Girouard MP, Begin C, Provencher V, Tremblay A, Boivin S, Lemieux S. Can we apply the dual-pathway model of overeating to a population of weight-preoccupied overweight women? *International Journal of Eating Disorders*. 2009;42(3):244-252. doi:https://dx.doi.org/10.1002/eat.20614
19. Gagnon-Girouard MP, Gagnon C, Begin C, et al. Couple dissatisfaction and eating profile: a mediation effect of coping style. *Eating and weight disorders : EWD*. 2010;15(4):e240-6.
20. Gerke CK, Mazzeo SE, Stern M, Palmberg AA, Evans RK, Wickham EP, 3rd. The stress process and eating pathology among racially diverse adolescents seeking treatment for obesity. *Journal of pediatric psychology*. 2013;38(7):785-93. doi:https://dx.doi.org/10.1093/jpepsy/jst042
21. Goossens L, Braet C, Bosmans G. Relations of dietary restraint and depressive symptomatology to loss of control over eating in overweight youngsters. *European child & adolescent psychiatry*. 2010;19(7):587-96. doi:https://dx.doi.org/10.1007/s00787-010-0089-5
22. Goossens L, Braet C, Van Vlierberghe L, Mels S. Loss of control over eating in overweight youngsters: the role of anxiety, depression and emotional eating. *European eating disorders review : the journal of the Eating Disorders Association*. 2009;17(1):68-78. doi:https://dx.doi.org/10.1002/erv.892
23. Hecht L, Haedt-Matt A, Schwartz N, Goldschmidt AB. Clinical Relevance of Overvaluation of Shape and Weight Among Bariatric Surgery Candidates. *Obesity surgery*. 2020;30(3):1163-1167. doi:https://dx.doi.org/10.1007/s11695-019-04372-8
24. Iannaccone M, D'Olimpio F, Cella S, Cotrufo P. Self-esteem, body shame and eating disorder risk in obese and normal weight adolescents: A mediation model. *Eating behaviors*. 2016;21:80-3. doi:https://dx.doi.org/10.1016/j.eatbeh.2015.12.010
25. Jankauskiene R, Baceviciene M. An exploration of the tripartite influence model of body image in Lithuanian sample of young adults: does body weight make a difference? *Eating and weight disorders : EWD*. 2021;26(6):1781-1791. doi:https://dx.doi.org/10.1007/s40519-020-00996-3
26. Kelly NR, Kosty D, Guerricabeitia L, Guidinger C, Williamson G. Evaluating components of existing theories for loss of control eating in a sample of young racially/ethnically diverse men. *Body image*. 2020;35:63-70. doi:https://dx.doi.org/10.1016/j.bodyim.2020.08.005
27. Kenny TE, Van Wijk M, Singleton C, Carter JC. An examination of the relationship between binge eating disorder and insomnia symptoms. *European eating disorders review : the journal of the Eating Disorders Association*. 2018;26(3):186-196. doi:https://dx.doi.org/10.1002/erv.2587
28. Lo Coco G, Sutton R, Tasca GA, Salerno L, Oieni V, Compare A. Does the Interpersonal Model Generalize to Obesity Without Binge Eating? *European eating disorders review : the journal of the Eating Disorders Association*. 2016;24(5):391-8. doi:https://dx.doi.org/10.1002/erv.2459
29. Marco JH, Canabate M, Martinez C, Banos RM, Guillen V, Perez S. Meaning in Life Mediates Between Emotional Deregulation and Eating Disorders Psychopathology: A Research From the Meaning-Making Model of Eating Disorders. *Frontiers in psychology*. 2021;12:635742. Erratum in: Front Psychol. 2022 Feb 15;13:849974 PMID: 35242091 [https://www.ncbi.nlm.nih.gov/pubmed/35242091]. doi:https://dx.doi.org/10.3389/fpsyg.2021.635742
30. Munir M, Dawood S. Weight stigma and disordered eating behaviors in Pakistani overweight adolescents: the mediating role of body esteem. *Eating and weight disorders : EWD*. 2021;26(6):1939-1948. doi:https://dx.doi.org/10.1007/s40519-020-01038-8
31. Palmeira L, Pinto-Gouveia J, Cunha M, Carvalho S. Finding the link between internalized weight-stigma and binge eating behaviors in Portuguese adult women with overweight and obesity: The mediator role of self-criticism and self-reassurance. *Eating behaviors*. 2017;26:50-54. doi:https://dx.doi.org/10.1016/j.eatbeh.2017.01.006
32. Palmeira L, Cunha M, Pinto-Gouveia J. The weight of weight self-stigma in unhealthy eating behaviours: the mediator role of weight-related experiential avoidance. *Eating and Weight Disorders*. 2018;23(6):785-796. doi:https://dx.doi.org/10.1007/s40519-018-0540-z
33. Palmeira L, Pinto-Gouveia J, Cunha M. The role of self-disgust in eating psychopathology in overweight and obesity: Can self-compassion be useful? *Journal of health psychology*. 2019;24(13):1807-1816. doi:https://dx.doi.org/10.1177/1359105317702212

34. Petersen JM, Durward C, Levin M. Weight-related psychological inflexibility as a mediator between weight self-stigma and health-related outcomes. *Bulletin of the Menninger Clinic*. 2021;85(3):316. doi:https://dx.doi.org/10.1521/bumc.2021.85.3.316
35. Rania M, Aloi M, de Filippis R, et al. Executive functions and depressive symptoms interplay in binge eating disorder: A structural equation model analysis. *European eating disorders review : the journal of the Eating Disorders Association*. 2021;29(5):811-819. doi:https://dx.doi.org/10.1002/erv.2854
36. Rayner KE, Schniering CA, Rapee RM, Hutchinson DM. A longitudinal investigation of perceived friend influence on adolescent girls' body dissatisfaction and disordered eating. *Journal of clinical child and adolescent psychology : the official journal for the Society of Clinical Child and Adolescent Psychology, American Psychological Association, Division 53*. 2013;42(5):643-56. doi:https://dx.doi.org/10.1080/15374416.2012.743103
37. Rice A, Lavender JM, Shank LM, et al. Associations among alexithymia, disordered eating, and depressive symptoms in treatment-seeking adolescent military dependents at risk for adult binge-eating disorder and obesity. *Eating and weight disorders : EWD*. 2022;doi:https://dx.doi.org/10.1007/s40519-022-01429-z
38. Shakory S, Van Exan J, Mills JS, Sockalingam S, Keating L, Taube-Schiff M. Binge eating in bariatric surgery candidates: The role of insecure attachment and emotion regulation. *Appetite*. 2015;91:69-75. doi:https://dx.doi.org/10.1016/j.appet.2015.03.026
39. Schaumberg K, Schumacher LM, Rosenbaum DL, et al. The role of negative reinforcement eating expectancies in the relation between experiential avoidance and disinhibition. *Eating Behaviors*. 2016;21:129-134. doi:https://dx.doi.org/10.1016/j.eatbeh.2016.01.003
40. Smith KE, Mason TB, Crosby RD, et al. State and trait positive and negative affectivity in relation to restraint intention and binge eating among adults with obesity. *Appetite*. 2018;120:327-334. doi:https://dx.doi.org/10.1016/j.appet.2017.09.020
41. Soulliard ZA, Brode C, Tabone LE, Szoka N, Abunnaja S, Cox S. Disinhibition and Subjective Hunger as Mediators Between Weight Bias Internalization and Binge Eating Among Pre-Surgical Bariatric Patients. *Obesity surgery*. 2021;31(2):797-804. doi:https://dx.doi.org/10.1007/s11695-020-05023-z
42. Wardle J, Waller J, Rapoport L. Body dissatisfaction and binge eating in obese women: the role of restraint and depression. *Obesity research*. 2001;9(12):778-87.
43. Wellman JD, Araiza AM, Solano C, Berru E. Sex differences in the relationships among weight stigma, depression, and binge eating. Psychological & Physical Disorders 3200. *Appetite*. 2019;133:166-173. doi:https://dx.doi.org/10.1016/j.appet.2018.10.029
44. Welsh DM, King RM. Applicability of the dual pathway model in normal and overweight binge eaters. *Body image*. 2016;18:162-7. doi:https://dx.doi.org/10.1016/j.bodyim.2016.06.007
45. Wooldridge JS, Herbert MS, Dochat C, Afari N. Understanding relationships between posttraumatic stress disorder symptoms, binge-eating symptoms, and obesity-related quality of life: the role of experiential avoidance. *Eating disorders*. 2021;29(3):260-275. doi:https://dx.doi.org/10.1080/10640266.2020.1868062
46. Zickgraf HF, Stefano E, Price J, Veldheer S, Rogers A, Rigby A. The relationship between food insecurity and binge and night eating symptoms in prebariatric surgery patients is mediated by depressive symptoms. *Surgery for obesity and related diseases : official journal of the American Society for Bariatric Surgery*. 2019;15(8):1374-1379. doi:https://dx.doi.org/10.1016/j.soard.2019.05.018
